# Molecular epidemiology and genome analysis of feline morbillivirus in household and shelter cats in Thailand

**DOI:** 10.1186/s12917-020-02467-4

**Published:** 2020-07-13

**Authors:** Surangkanang Chaiyasak, Chutchai Piewbang, Anudep Rungsipipat, Somporn Techangamsuwan

**Affiliations:** 1 Department of Pathology, Faculty of Veterinary Science, Chulalongkorn University, Bangkok, 10330 Thailand; 2 Animal Virome and Diagnostic Development Research Group, Faculty of Veterinary Science, Chulalongkorn University, Bangkok, 10330 Thailand

**Keywords:** Feline morbillivirus, Fusion, Hemagglutinin, Phosphoprotein, Selective pressure analysis, Urine

## Abstract

**Background:**

Feline morbillivirus (FeMV) has been discovered in domestic cats associated with tubulointerstitial nephritis, but FeMV is also detected in healthy cats. This research aimed to identify and characterize the FeMV strains detected in a Thai cat population.

**Results:**

Two-hundred and ninety-two samples (131 urine and 161 blood) derived from 261 cats (61 sheltered and 200 household cats) were included for investigating the FeMV prevalence using real-time reverse transcription PCR. The overall prevalence of FeMV detection was 11.9% (31/261) among both samples, which accounted for 14.5% (19/131) and 7.5% (12/161) of the urine and blood samples, respectively. Among the FeMV-PCR positive cats, the FeMV-detected prevalence was insignificantly associated with healthy cats (58.1%; 18/31) or urologic cats (41.9%; 13/31). Full-length genome analysis of these FeMV-Thai strains revealed that their genomes clustered together in the FeMV-1A clade with up to 98.5% nucleotide identity. Selective pressure analysis showed that overall FeMV-1 has undergone negative selection, while positive selection sites were more frequently observed in the phosphoprotein gene.

**Conclusions:**

The detected FeMV infections in the Thai cat population were not correlated with urologic disorders, although the virus was more detectable in urine samples. The genetic patterns among the FeMV-1 Thai strains were more consistent. A large-scale study of FeMV in Thai cat samples is needed for further elucidation.

## Background

Feline morbillivirus (FeMV), belonging to genus *Morbillivirus*, family *Paramyxoviridae*, is a 16,050-bp length, non-segmented, enveloped, single-stranded, negative-sense RNA virus, that encodes for six genes; nucleocapsid (N), phosphoprotein (P/V/C), matrix (M), fusion (F), hemagglutinin (H), and RNA polymerase (L) [1]. Among the functional proteins, the H and F glycoproteins on the viral membrane play a key role for attaching and fusing the host cells membrane, respectively [2]. Additionally, the diversity of H gene characterization is potentially affected the host range and virulence [3–6]. Since the first identification of FeMV in domestic cats showing tubulointerstitial nephritis in Hong Kong in 2012 [[1], the virus has been investigated in both clinically healthy and ill cats in many countries, such as Japan, Turkey, Germany, Italy, USA, Brazil, and Malaysia [7–16]. The prevalence of FeMV detection ranges from 0.2–40% based on the tested samples, comprised such as blood, urine, rectal swab, fresh tissues, and formalin-fixed paraffin-embedded tissues [7–10, 12, 14–16]. Because the first emergence of FeMV was associating with renal disease, the initial studies on FeMV identification were conducted in urine samples, while comparison of FeMV detection in urine and other derived samples was also reported [1, 9]. Geographically, the prevalence of FeMV-positive samples is seemingly inconsistent, with a higher detection rate in Japan (ranging from 6.1–23.1%) [9, 13, 17], Italy (ranging from 1.2–31.8%) [8, 18], and Malaysia (50.8%) [12], while a lower detection rate was reported in the USA, Germany, Brazil, and Turkey [7, 14–16, 18].

Currently, full-length genome analysis of FeMV has categorized this virus into the two genotypes of FeMV-1 (former FeMV) [19, 20] and FeMV-2 (former FeMV-GT2), the latter of which was recently detected in cats showing urinary tract disease (UTD) in Germany [19]. The FeMV-1 genotype was subsequently clustered based on the partial L gene sequence into the FeMV-1A, −1B and -1C subgroups [21]. However, neither the FeMV-1 nor FeMV-2 genotype is able to clarify the association with nephropathy in cats [7, 11, 13, 14, 16, 19]. Therefore, the genetic characteristics of FeMV in many regions remain to be elucidated for studying viral pathogenesis, such as different cellular tropism [19, 22].

Since most RNA viruses are prone to mutation, due to the lack of an internal proof-reading mechanism during replication that results in a high rate of variant nucleotide substitutions, the identification of local strains would be beneficial for the further future management of disease control and monitoring. The genetic characterization of the newly identified FeMV in Thailand would contribute to the basic knowledge of not only the identification of FeMV but also on its evolution. Thus, this study aimed to establish the identification of FeMV Thai strains with viral genetic characterizations. Molecular genetic recombination and selective pressure analysis for evolutionary evaluation of the FeMV were also investigated. This fundamental data on the molecular epidemiology of FeMV strains might contribute to a more comprehensive understanding of FeMV evolution.

## Results

### Detection of FeMV in the urine and blood of Thai cats

The presence of FeMV in all the samples was 11.9% (31/261), as detected by the RT-qPCR assay. Among the samples, the FeMV was detected at a two-fold higher rate in the urine (19/131, 14.5%; as six shelter and 13 household cats) than in the blood (12/161, 7.5%; all were shelter cats). However, from the 61 shelter cats examined, although 18/61 (29.5%) showed FeMV in either the blood or the urine, no any cat showed FeMV-positive results in both sample types at the same time (Tables 1 and 2).

**Table 1:** Positivity rate of FeMV detection by RT-PCR

		Shelter cats (*n* = 61)		Group A Household cats (*n* = 100)	Group B Household cats (n = 100)	Total cats (*n* = 261)	
		Urine (*n* = 31)	Blood (n = 61)	Urine (n = 100)	Blood (n = 100)	Urine (*n* = 131)	Blood (*n* = 161)
FeMV-PCR positive	Individual sample type	6^a^	12^a^	13^b^	0	19/131 (14.5%)	12/161 (7.5%)
	Average	18/61 (29.5%)		13/200 (6.5%)		31/261 (11.9%)	

^a^ There was no positive FeMV-PCR result in the same shelter cat

^b^ All FeMV-PCR positive cats showed abnormal urinalysis results, representing urologic disorders

**Table 2:** Biological data of cats with FeMV-positive results

Sample type	Sample No. ^a^	Sex ^b^	Clinical sign ^c^	Rapid test ^d^	
				FeLV Ag	FIV Ab
Urine	U16	F (n)	CI	–	–
	U36	M (n)	CI	–	–
	U44	F (n)	CI	–	–
	U48	M (n)	CI	–	–
	U50	F (n)	CI	–	–
	U55	F (n)	CI	–	–
	CTL-8	M (n)	UTD	n/a	n/a
	CTL-15	M (n)	UTD	n/a	n/a
	CTL-16	M (n)	UTD	n/a	n/a
	CTL-25	n/a	UTD	n/a	n/a
	CTL-32	M	UTD	n/a	n/a
	CTL-43	M	UTD	n/a	n/a
	CTL-58	M	UTD	n/a	n/a
	CTL-60	M	CKD + DM	n/a	n/a
	CTL-63	M	UTD	n/a	n/a
	CTL-70	n/a	UTD	n/a	n/a
	CTL-89	n/a	UTD	n/a	n/a
	CTL-90	M	UTD	n/a	n/a
	CTL-100	n/a	CKD	n/a	n/a
Blood	E11	F (n)	CI	–	–
	E20	F (n)	CI	–	–
	E23	F (n)	CI	–	–
	E25	M (n)	CI	–	–
	E27	F (n)	CI	–	–
	E29	M (n)	CI	+	–
	E32	F (n)	CI	–	–
	E49	M (n)	CI	–	–
	E51	M (n)	CI	–	–
	E53	M (n)	CI	–	–
	E56	F (n)	CI	–	–
	E61	F (n)	CI	–	+

^a^ U = urine from shelter cat, CTL = urine from household cat, E = blood from shelter cat

^b^ F = female, M = male, (n) = neutered, n/a = no data available

^c^ CI = clinically insignificant, CKD = chronic kidney disease, DM = diabetes mellitus, UTD = urinary tract disease

^d^ FeLV Ag = feline leukemia virus antigen, FIV Ab = feline immunodeficiency virus antibody, − = negative, + = positive

Considering the category of tested cats, the presence of FeMV detection in the shelter cats (18/61, 29.5%) was 4.5-fold higher than in the household cats (13/200, 6.5%). For the FeMV-positive urine samples, FeMV infection was found in cats with UTD (11/19, 57.9%; all were household cats), followed by no clinical significance or apparently healthy (6/19, 31.6%; all were shelter cats) and chronic kidney disease (CKD) (2/19, 10.5%; both were household cats) (Table 2).

### Association between the FeMV infection status, urine characteristics, urologic diseases and feline retrovirus detection

The urinalysis of the group A household cats was determined in terms of the physical, chemical, and microscopic features and tabulated according to the FeMV-PCR positive results. Even though there were no statistical significances between the presence of FeMV in the urine and the urine characteristics (*P* > 0.05), some features, such as hematuric, pyuric, proteinuric, and aciduric urines, had a higher numerical tendency to be positive with FeMV detection. Among the 91 urine samples with urologic conditions from 100 household cats examined, the positive FeMV in the urine was evidenced at a six-fold lower rate (13/91, 14.3%), without significance, than the negative FeMV counterpart (78/91, 85.7%) (*P* > 0.05, Supplementary Table S2). Moreover, among the FeMV-PCR positive cats, only one shelter cat each revealed a positive FeLV antigen (no. E27) and a FIV antibody (no. E61) (Table 2).

### Phylogenetic analysis and genome organization of the full-length FeMV-Thai strains

Three full-length FeMV-Thai strains were obtained and have been submitted to GenBank as U16–2016 (MF627832; 16,050 nt length), CTL16–2018 (MN164531; 15,946 nt length), and CTL43–2018 (MN164532; 15,949 nt length). After nucleotide alignment and analysis, all three FeMV-Thai strains displayed the six consecutive gene sequences (N-P-M-F-H-L), comprised of the N (1,560 nt), P (1,476 nt), M (1,014 nt), F (1,632 nt), H (1,788 nt), and L (6,609 nt) genes, which encoded for 520, 492, 338, 544, 596, and 2203 deduced amino acids, respectively. The different genome lengths among these three FeMV-Thai strains were due to the incomplete nucleotide sequence at the 5′ end of CTL16–2018 and CTL43–2018.

**Fig. 1:**
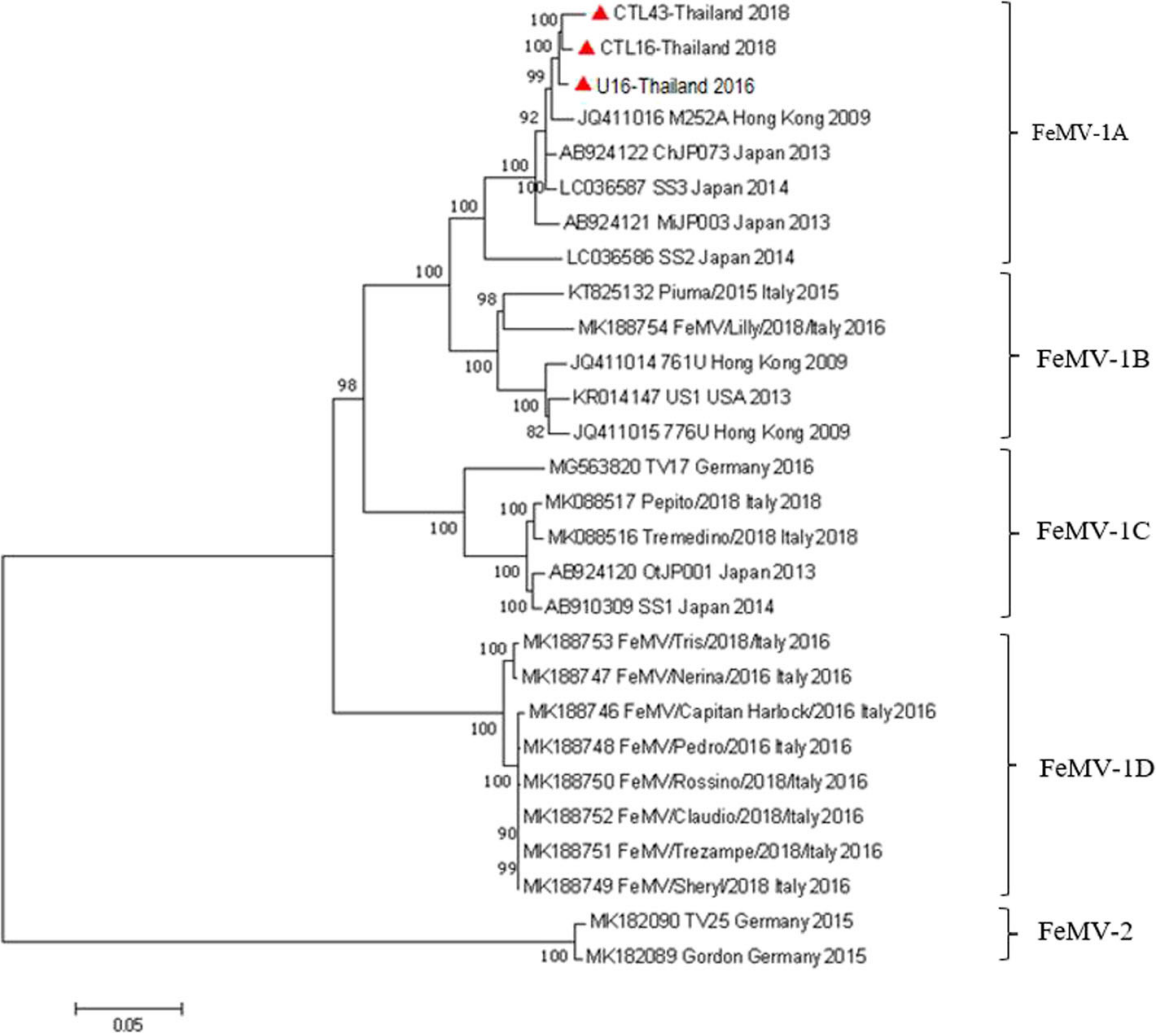
Phylogenetic analysis of the full-length genome sequence of FeMV strains. Scale bar is the substitution rate per site. The ML method with GTR model and 1000 bootstrap replications (shown as % value) were performed in the Mega 7 software. The FeMV strains were grouped into two genotypes and subdivided into four clades of FeMV-1, the same topology as the phylograms from each gene. Red triangles show the three Thai FeMV isolates of this study

Phylogenetic analysis of the full-length genome of the three FeMV-Thai strains revealed that they shared the same monophyletic topology of the FeMV-1 genotype and segregated into the FeMV-1A clade, clustered with the FeMV strains reported from Hong Kong (M252A) and Japan (strain ChJP073, MiJP003, SS2, and SS3) (Fig. 1). Pairwise nucleotide identity analyses revealed that these FeMV-Thai strains had the highest nucleotide identity to the FeMV-1A strain SS3 (97.8–98.5%), and to a lesser extent, to the FeMV-1A strain M252A (97.5–98.3%). Likewise, comparisons of nucleotide identities between three FeMV-1A Thai strains and the other FeMV clades displayed relatively high percentage similarities with the strains from FeMV-1B (91.3–92.0%), FeMV-1C (87.7–88.3%), FeMV-1D (86.9–87.4%), and FeMV-2 (81.6–81.9%) (Supplementary Table S3).

For the individual nucleotide and deduced amino acid analysis between these three FeMV-1A Thai strains and the FeMV-2 genotype (Gordon strain), the result showed that the P segment contained the most divergent nucleotide and amino acid identity at 80.4–80.6% and 69.2–74.5%, respectively, while the L segment revealed the most conserved nucleotide and amino acid identity at 82.4–82.5% and 90.6–90.8%, respectively (Supplementary Table S4). The phylogenetic trees constructed from each gene of FeMV-Thai strains revealed consistent topologies to the tree constructed from the full-length genome, in presenting the homology cluster in FeMV-1A (Supplementary Figs. S1–3).

### Unique deduced amino acid residues of the F and H genes among the FeMV genotype and clade

Seven FeMV samples (U16–2016, CTL15–2018, CTL16–2018, CTL25–2018, CTL32–2018, CTL43–2018, and CTL58–2018) were further sequenced for the complete coding region of the F and H genes, and these have been deposited in GenBank with accession no. MF627832 (full length genome of U16–2016), and MN316616–21 and MN316622–7 for the F and H genes, respectively, of the other six CTL derived samples.

Interestingly, we found that the deduced amino acid residues of the start codon peptides of the F gene were distinguishable among the FeMV genotype and clades. The deduced start codon peptides of the FeMV-1A Thai strains, from this study, presented as MNRIG (U16–2016) and MNRIR (other six CTL derived samples), which were different from the previous FeMV-1A strains from Hong Kong (M252A) and Japan (SS2, SS3, and ChJP073) that were MNRIK. Moreover, the overall deduced amino acids of the F gene for the FeMV-Thai strains were identical to the other FeMV-1 genotype, except for residues 71 and 99 (Table 3). For the H gene, the amino acid residues of FeMV-1A in this study were identical to other previous FeMV-1 strains at positions that were distinguishable from FeMV-2, such as residues 6, 17, 51, 58, 68, etc. Nevertheless, the residues 75, 82, 129, 500, 542, and 561 were diverse from FeMV-1B to -1D (Table 4).

**Table 3:** Differences in start codon peptides and deduced amino acid residues of the FeMV F gene

FeMV	Start codon peptide	Amino acid residues											
		10	11	71	99	190	286	372	373	374	491	510	522
FeMV-1A	MNRIR MNRIG MNRIK	S	S	I	V	S	T	L	T	K	L	Y	T
FeMV-1B	MNRIK	S	S	T	A	S	T	L	T	K/E	L	Y	T
FeMV-1C	MGKIK	S	S	M/I	A/T	S	T	L	T	K	L	Y	T
FeMV-1D	MDKIK	S	S	I	T	S	T	L	T	K	L	Y	T
FeMV-2	MYKIK	G	F	A	T	A	V	L	I	N	S	C	A

**Table 4:** Differences in the deduced amino acid residues of the FeMV H gene

FeMVs	Amino acid residues																				
	6	17	51	58	68	75	77	82	129	132	192	257	295	349	420	496	500	542	546	561	595
FeMV-1A	I	G	I	T	T	R	E	Q	V	D	K	V	L	I	A	K	T	L	K	S	K
FeMV-1B	I	G	I	T	T	R	E	Q	V	D	K	V	L	I	A	K	T	V/I	K	S	K/Q
FeMV-1C	I	G	I	T	T	R	E	H	V	D	K/E	V	L	I	A	K	M	I	K	N	K
FeMV-1D	I	G	I	T	T	K	E	Q	M	D	K	V	L	I	A	K	T	I	K	N	K
FeMV-2	N	S	T	I	M	Q	K	K	T	N	N	T	P	M	E	I	L	V	R	A	N

### Recombination and selective pressure analysis

To further investigate the possible evolution of the FeMV-Thai strains, we performed recombination analysis on the complete genome of the FeMV-Thai strains and, in particular, on the complete F and H gene of FeMV-1, with the other strains available in GenBank using the RDP method. After all models were implemented, no evidence of putative recombination breakpoint was found in our study.

To overcome the selective-pressure mediated altered evolution of FeMV, this study explored the selective pressure analysis on each gene of FeMV using various statistical methods. Overall, the FeMV evolution was found to have undergone negative selective pressure, but positive selection sites were presented in the P, N, H, and F genes. Among the six genes of FeMVs, the P gene showed the highest frequency of potential positive selection sites (9/492 sites using the FEL model) at amino acid sites 58, 64, 80, 88, 132, 154, 156, 218, and 249. This was followed by the H gene (4/596 sites using MEME model) at amino acid sites 62, 86, 104 and 170, N gene (4/520 sites using the MEME model) at amino acid sites 6, 8, 10 and 132, F gene (1/544 site using the MEME model) at amino acid site 503, based upon a dN/ dS > 1 and a *p*-value of < 0.1. In contrast, the M and L genes revealed a negative selection pressure with a dN/ dS < 1.

## Discussion

Although FeMV has been recognized in CKD cats since 2012 [1] and gained wide attention worldwide, many interesting points, including its pathogenesis, cellular tropism, and association of CKD pathology, through its evolution for viral adaptation are still unclear and in need of further investigation. This study is the first report of the molecular identification and epidemiology of FeMV-1 in Thailand, revealing the existence of FeMV in Thai cats. The prevalence of FeMV in the present study was 11.9%, which was quite similar to that reported from Hong Kong (12.3%) [1], but higher than those reported in some other regions, such as Turkey (5.4%) [16] and Japan (6.1%) [9], or lower than that in Malaysia (39.4%) [12]. This discrepancy in the FeMV prevalence may result from various factors, such as the cat’s lifestyle (household/stray/shelter/chance of street access), habitat areas (urban/suburban/rural), and testing groups [9, 16].

In this study, the urine samples provided an almost two-fold higher FeMV detection rate compared to the blood samples (14.5 and 7.5%, respectively). Furthermore, FeMV RNA was not detected in the blood from cats with FeMV-positive urine. This finding was consistent with a previous report [16] and may suggest that FeMV-positive cats probably has the result of an ongoing acute infection as found as in other morbilliviruses [23]. Thus, the possibility of detectable FeMV in cats might be increased if various biological samples were tested from the same cat. Due to the fact that most FeMV identifications underwent detection of the virus in either the urine, kidney, or both samples, this might underestimate the true FeMV prevalence in regions [7, 11, 15, 16, 18, 21]. Currently, there have been few studies and minimal case number testing of FeMV RNA in blood samples [9, 16, 24], suggesting a low prevalence of FeMV RNA in blood samples. Conversely, the relative high number of FeMV RNA blood-positive cats (12/61) in our study might be due to the tested cats, which had close contact among other cats in the shelter, leading to persistent viral circulating in the shelter over time.

Since the FeMVs were frequently detected in the urine, previous studies have attempted to figure out the relationship between the presence of FeMV and the pathology of the kidneys [1, 8, 19, 25]. However, there were few reports about a correlation between FeMV infection (identification) and urinalysis parameters [15, 26]. Here, we provided another attempt to investigate the correlation of the presence of FeMV and urine parameters, and found that the household cats positive for FeMV in their urine cats were associated with abnormal urine characteristics, such as hematuria, pyuria, proteinuria, and aciduria. Even though this association was not statistically significant, it might raise a note of FeMV infection in cats showing urinary tract infections (UTIs), since a small number of recent publications have demonstrated urinalysis results in cats with FeMV [7, 16, 18]. However, other potential pathogens contributing to the feline UTIs, which are bacteria, FIV, leptospirosis and bartonellosis [27], were not excluded from this study. Therefore, the bacteriology examination or FIV detection should be warrant in future study to evaluate the single FeMV infection or the coinfection with other infectious agents in cats with UTI.

In the present study, the identified Thai FeMV isolates had a genetic homology amongst themselves and were classified as FeMV-1A without any evidence of genetic recombination. This finding may indicate that the local FeMV-1 strain has been circulating in Thai cat populations. Since the H and F genes of other morbilliviruses, notably the CDV, have played crucial roles associating cellular tropism and host membrane fusion, antigenic and sequence variations may alter the virulence of the virus [4]. Furthermore, the antigenic variation of the CDV H gene is used as geographic signature to identify the origin of the CDV, resulting in widely used for lineage classification [3, 28–30]. Therefore, the genetic variations of the H and F genes of the FeMVs have been focused and interpreted in this study. Contrast to the CDV, we observed that the F gene of the FeMVs revealed more hypervariable portion than the H gene. The effects of amino acid mutations in the F and H genes, which are hypothesized to be associated with the viral infectivity and virulence, needs further investigation in a future study. In addition, we also proposed distinct deduced amino acid residues in the F and H genes that can potentially differentiate isolates within the FeMV clade.

RNA viruses possess a high mutation rate as a result of viral RNA polymerases lack a proof-reading property, then allowing rapid adaptations to various selection pressures [28, 31]. Moreover, recent studies have shown evidence of other morbilliviruses undergoing selective pressure, such as the negative and positive selective pressure for canine distemper virus and measles virus, respectively [3, 32]. In this study, we found that overall the FeMV evolution has undergone a negative selective pressure, but positive selection sites were observed, with the highest frequency in the P gene, followed by in the H, N and F genes. This finding may suggest that these FeMV genes may play a role in FeMV evolution and emphasize the importance of P gene, the non-structural gene of morbillivirus, in the aspect of immunopathogenesis in particular hosts as already mentioned previously in rinderpest virus (RPV) and measles virus (MeV) [33]. However, the data used in this study were restricted to the 23 currently available full-length FeMV genomes. More analyzed sequences would likely allow a more clear understanding of FeMV evolution.

## Conclusions

This study presented the first report of FeMV-1 identification in domestic cats in Thailand with a higher detection rate in the urine than in blood samples. The FeMV was detected in both urological-ill and healthy cats without any association, although a higher detection rate was found in healthy cats. Genetic analysis of FeMV-Thai strains revealed a high genetic homology among the Thai strains, which were clustered in the FeMV-1A clade. Without any evidence of genetic recombination, the FeMV has undergone evolution by negative selection but with some positive selection sites in the P, H, N and F genes. Further study of FeMV identification at a larger scale and different sample groups is necessary.

## Methods

### Animals and sample collection

Sixty-one cats from shelters locating in Saraburi province and 200 cats from different households in Bangkok (both are located in central Thailand, about 100 km apart) were included in this study. A total of 292 samples, comprised of 131 urine (derived from 31 shelter and 100 household cats) and 161 EDTA anticoagulated blood samples (derived from 61 shelter and 100 household cats) were collected during 2016 to 2018. Of note, the urine and blood samples were parallelly collected from 31 shelter cats, whereas individual urine or blood samples were collected from 100 household cats each (designated as group A and B) (Table 1). Signalment and clinical presentations were recorded by veterinarians who collected the samples for further analysis.

### Urinalysis and feline retrovirus infection screening

Blood samples (where available) were tested for feline immunodeficiency virus (FIV) antibody and feline leukemia virus (FeLV) antigen using a commercial test kit (Bionote, Gyeonggi-do, South Korea). Routine urinalysis, including physical (color and transparency) and chemical (nitrite, pH, glucose, protein, occult blood, ketone, urobilinogen, leukocytes, ascorbic acid, and bilirubin) characteristics, was performed on the 100 urine samples from group A using URIT 11G test strips (URIT®, China). The specific gravity of the urine was measured using a refractomerter. All samples were then kept at − 80 °C until further used.

### Two-stage real-time reverse transcription polymerase chain reaction (RT-qPCR)

Viral nucleic acid was extracted from 200 μL of urine or blood samples using a Viral Nucleic Acid Extraction Kit II (GeneAid, Taipei, Taiwan) according to manufacturer’s recommendation. The RNA concentration was qualified and quantified using a NanoDrop Lite Spectophotometer (Thermo Fisher Scientific Inc., Waltham, MA, U.S.A.). For the first stage RT-qPCR, complementary DNA (cDNA) was constructed from 100 ng of extracted RNA using Omniscript Reverse Transcription Kit (Qiagen GmbH, Hilden, Germany), following the manufacturer’s protocol. The derived cDNA was kept at − 20 °C until further used.

For the second stage RT-qPCR, the presence of FeMV in each sample was detected using a KAPA SYBR fast qPCR master mix (2X) universal (KAPABIOSYSTEMS, Sigma-Aldrich®, Modderfontein, South Africa) with specific primer pairs targeting the L gene of FeMV, as previously described [1]. The qPCR reaction was performed on Rotor-Gene Q real-time PCR cycler (Qiagen GmbH, Manheim, Germany) with 40 cycles of 95 °C for 3 s, 60 °C for 20 s, and 72 °C for 20 s acquiring fluorescence A green. The software reporting cycling A green and melt A green compared that for the positive control (courtesy of Prof. Furuya) and no template control. Subsequently, selected positive RT-qPCR samples were resolved by 1.5% (w/v) agarose gel electrophoresis, purified using NucleoSpin Extract II kit (Macherey-Nagel, Düren, Germany), and submitted for commercial bidirectional Sanger’s sequencing (Macrogen Inc., Incheon, South Korea) to confirm the presence of FeMV.

### Amplification and sequencing of the complete F and H genes, and full-length genome of Thai FeMV

The seven FeMV-PCR positive samples (U16–2016, CTL15–2018, CTL16–2018, CTL25–2018, CTL32–2018, CTL43–2018, and CTL58–2018) were further amplified for the complete F and H genes by RT-PCR assays with a set of specific primers targeting the F and H genes. These primers were designed from the alignment of various FeMV strains available in GenBank (Supplementary Table S1). Furthermore, the samples of U16–2016, CTL16–2018, and CTL43–2018 were subjected to full-length genome sequencing using the panel of primers described previously [17] with some modifications. Positive amplicons were purified and subsequently submitted for Sanger’s sequencing as mentioned above. Derived sequences were then aligned to construct the whole genome of these three FeMV-Thai strains.

### Genetic characterization and phylogenetic analysis of the FeMV Thai strains

The obtained nucleotide sequences of the FeMV-Thai strains, both the full-length genome and the individual genes, were constructed by BioEdit Sequence Alignment Editor Version 7.2.5 and compared to other previously published FeMV strains retrieved from GenBank. Phylogenetic analyses of detected FeMVs were performed using the maximum likelihood (ML) method implemented in the MEGA7 software package version 7.0. Genetic topology tree was constructed using the general-time reversible model (GTR) as the best-fit model of nucleotide substitution, according to the Bayesian information criterion, with 1000 bootstrapped replicates. Pairwise distance of the FeMV genome was calculated using the compute pairwise distance in MEGA7. Deduced amino acid sequences analysis of the F and H genes were performed using the BioEdit software.

### Recombination and selective pressure analyses

To detect any potential recombination sites in the FeMV-Thai strains, a panel of previously described statistical methods was applied [32]. Briefly, each recombination analysis, including RDP, GENECONV, BootScan, MaxChi, Chimaera, SiScan, and 3Seq, were run with default settings in the Recombination Detection Program (RDP) package version 4.0, and were performed on the alignment of FeMV sequences. Any potential breakpoint signals detected by at least four models (all with *P* < 0.01) were considered to ensure the positive recombination breakpoints [34].

To determine the frequency of selective pressure breakpoints of FeMV-Thai strains, the panel tests were run on six individual genes (N, P, M, F, H, and L). Non-neutral selection of nucleotide substitutions was calculated using the ratio value between nonsynonymous (dN) and synonymous (dS) substitutions with a ML approach, reconstructed using the Datamonkey web server (http://www.datamonkey.org). Single-likelihood ancestor counting, Fixed-effects likelihood (FEL), Mixed Effect Model Evolution (MEME), and Fast, Unconstrained Bayesian AppRoximation (FUBAR) approaches were used, accepting significance at the *P* ≤ 0.1 level in all the methods. The Bayes factor was set at 50 to estimate the rate of dN and dS within an individual codon [35]. Positive, neutral, and negative selections were defined as dN/dS > 1, dN/dS = 1, and dN/dS < 1, respectively.

### Statistical analysis

The associations between the FeMV status (positive or negative), category of cats (shelter or household), and type of samples (urine or blood) were tested with odds ratio. The presence of FeMV in the urine were analyzed with the urine characteristics from urinalysis, and wtih the urologic associated diseases using Chi-square test or Fisher’s exact test (Prism 6, GraphPad), with a 95% confidence interval and accepting significance at the *P* < 0.05 level.

## Supplementary information

**Additional file 1: Table S1**. Primers for the RT-PCR amplification of the FeMV F and H gene. **Table S2**. Urinalysis and FeMV RT-PCR results from 100 cats’s urine samples. **Table S3.** Nucleotide identity of the full-length genome between FeMV-Thai strains and other genotypes and clades. **Table S4.** Nucleotide and amino acid identities of each gene between the FeMV-Thai strains (FeMV-1) and the Gordon strain (FeMV-2). **Figure S1.** Phylogenetic analysis of the codon region sequence of the six genes in FeMV strains. **Figure S2.** Phylogenetic tree of the codon region of the F gene among FeMVs. Scale bar is the substitution rate per site. The ML method with a GTR model and 1000 bootstrap replicates (shown as a %) were performed in the Mega 7 software. **Figure S3.** Phylogenetic tree of the codon region of the H gene among FeMVs. Scale bar is the substitution rate per site. The ML method with a GTR model and 1000 bootstrap replicates (shown as a %) were performed in the Mega 7 software.

## Data Availability

All the data supporting our findings is contained within the manuscript. Sequences from this study have been deposited in NCBI GenBank under accession numbers as followed: three full-length FeMV-Thai strains U16–2016 (MF627832), CTL16–2018 (MN164531), and CTL43–2018 (MN164532); six complete coding region of the F and H genes of FeMV-Thai strains CTL15–2018, CTL16–2018, CTL25–2018, CTL32–2018, CTL43–2018, and CTL58–2018 with accession no. MN316616–21 and MN316622–7 for the F and H genes, respectively.

## Abbreviations

FeMV: Feline morbillivirus
RT-qPCR: Real-time reverse transcription polymerase chain reaction
cDNA: complementary DNA
UTD: Urinary tract disease
UTIs: Urinary tract infections
FeLV: Feline leukemia virus
FIV: Feline immunodeficiency virus
N: Nucleocapsid
P/V/C: Phosphoprotein
M: Matrix
F: Fusion
H: Hemagglutinin
L: RNA polymerase
dN/dS: ratio value between nonsynonymous and synonymous
FeL: Fixed-effects likelihood
MEME: Mixed Effect Model Evolution
FUBAR: Fast, Unconstrained Bayesian AppRoximation
