# Supplementary material for: Molecular epidemiology and genome analysis of feline morbillivirus in household and shelter cats in Thailand
Source: BMC Vet Res. 2020 Jul 13;16:240. doi: 10.1186/s12917-020-02467-4 (PMC7359279; doi:10.1186/s12917-020-02467-4)
Supplement: Supplementary file 1 — Additional file 1: Table S1. Primers for the RT-PCR amplification of the FeMV F and H gene. Table S2. Urinalysis and FeMV RT-PCR results from 100 cats’s urine samples. Table S3. Nucleotide identity of the full-length genome between FeMV-Thai strains and other genotypes and clades. Table S4. Nucleotide and amino acid identities of each gene between the FeMV-Thai strains (FeMV-1) and the Gordon strain (FeMV-2). Figure S1. Phylogenetic analysis of the codon region sequence of the six genes in FeMV strains. Figure S2. Phylogenetic tree of the codon region of the F gene among FeMVs. Scale bar is the substitution rate per site. The ML method with a GTR model and 1000 bootstrap replicates (shown as a %) were performed in the Mega 7 software. Figure S3. Phylogenetic tree of the codon region of the H gene among FeMVs. Scale bar is the substitution rate per site. The ML method with a GTR model and 1000 bootstrap replicates (shown as a %) were performed in the Mega 7 software. [file 12917_2020_2467_MOESM1_ESM.docx]

**Supplementary Table S1**

Primers for the RT-PCR amplification of the FeMV F and H gene.

| **Primer name** | **Primer sequence (5’-3’)** | **Nucleotide position**  **(based on MF627832)** | **Product size**  **(bp)** |
| --- | --- | --- | --- |
| CFW-F1 | ATACCAATGGTCTCTTCAAGATTA | 4374–4397 | 753 |
| CRV-F1 | TTGGGAACCATCTTTATAACCAT | 5104–5126 |  |
| CFW-F2 | GGRGTTATAAGTACTAAGCA | 5044–5063 | 714 |
| CRV-F2 | CAAATCAGTCATATCTGCAT | 5738–5757 |  |
| CFW-F3 | AATGGTAATCTTCAGGCA | 5641–5658 | 893 |
| CRV-F3 | AGCGGTTCAATTGAAGTA | 6516–6533 |  |
| CFW-H1 | GTTAGAGCAATCAGATAAGATT | 6354–6375 | 926 |
| CRV-H1 | GTAGGAATATGAAGTCCTA | 7261–7279 |  |
| CFW-H2 | ACTTAGGAATCCATGTTAAT | 6929–6948 | 768 |
| CRV-H2 | ACTCGATATTGAACATCAGT | 7677–7696 |  |
| CFW-H3 | TCAGCAATCAACGTATAACATT | 7600–7621 | 664 |
| CRV-H3 | AGAGAATTATGAGATGGAGCT | 8243–8263 |  |
| CFW-H4 | GACAACTCTGAGAATTACTGTA | 8097–8118 | 816 |
| CRV-H4 | TTGGTAGTCTGACTGCTC | 8895–8912 |  |

**Supplementary Table S2**

Urinalysis and FeMV RT-PCR results from 100 cats’s urine samples.

| Urinalysis | FeMV-positive | FeMV-negative | *P*-value |
| --- | --- | --- | --- |
| *- Clarity* |  |  |  |
| Turbid | 4 | 33 | 0.6179 |
| Clear | 9 | 54 |  |
| *- Specific gravity* |  |  |  |
| Hyposthenuria | 1 | 13 | 0.528 |
| Isosthenuria | 9 | 46 |  |
| Hypersthenuria | 3 | 28 |  |
| *- pH* |  |  |  |
| Aciduria | 9 | 61 | 0.9483 |
| Alkalinuria | 4 | 26 |  |
| *- Hematuria* |  |  |  |
| Positive | 11 | 56 | 0.1476 |
| Negative | 2 | 31 |  |
| *- Pyuria* |  |  |  |
| Positive | 10 | 57 | 0.4146 |
| Negative | 3 | 30 |  |
| *- Proteinuria* |  |  |  |
| Positive | 9 | 43 | 0.1825 |
| Negative | 4 | 44 |  |
| *- Crystalluria* |  |  |  |
| Positive | 2 | 20 | 0.537 |
| Negative | 11 | 67 |  |
| *- Bilirubinuria* |  |  |  |
| Positive | 0 | 2 | 0.583 |
| Negative | 13 | 86 |  |
| *- Glucosuria* |  |  |  |
| Positive | 1 | 3 | 0.4664 |
| Negative | 12 | 84 |  |
| *- Ketonuria* |  |  |  |
| Positive | 0 | 3 | 0.4966 |
| Negative | 13 | 84 |  |
| *- Urologic disease* |  |  |  |
| Yes | 13 | 78 | 0.6007 |
| No | 0 | 9 |  |

**Supplementary Table S3**

Nucleotide identity of the full-length genome between FeMV-Thai strains and other genotypes and clades.

| **FeMV-Thai strains** | **Nucleotide identity (%)** | | | | | | | | | |
| --- | --- | --- | --- | --- | --- | --- | --- | --- | --- | --- |
|  | **FeMV-1A** | | **FeMV-1B** | | **FeMV-1C** | | **FeMV-1D** | | **FeMV-2** | |
|  | **SS3** | **M252** | **US1** | **Piuma/2015** | **TV17** | **OtJP001** | **Tris/2018** | **Shery/2018** | **TV25** | **Gordon** |
| **U16-2016** | 98.5 | 98.3 | 91.9 | 92.0 | 88.2 | 88.3 | 87.4 | 87.4 | 81.8 | 81.9 |
| **CTL16-2018** | 98.4 | 98.08 | 91.8 | 91.9 | 88.1 | 88.2 | 87.2 | 87.2 | 81.8 | 81.9 |
| **CTL43-2018** | 97.8 | 97.53 | 91.3 | 91.5 | 87.7 | 87.8 | 86.9 | 86.9 | 81.6 | 81.7 |

**Supplementary Table S4**

Nucleotide and amino acid identities of each gene between the FeMV-Thai strains (FeMV-1) and the Gordon strain (FeMV-2).

| **FeMV-Thai strains** | **Nucleotide and amino acid identities (%)** | | | | | | | | | | | | |
| --- | --- | --- | --- | --- | --- | --- | --- | --- | --- | --- | --- | --- | --- |
|  | **N gene** | | **P gene** | | **M gene** | | **F gene** | | **H gene** | | **L gene** | | |
|  | **nt** | **aa** | **nt** | **aa** | **nt** | **aa** | **nt** | **aa** | **nt** | **aa** | **nt** | **aa** |  |
| **U16-2016** | 81.9 | 90.2 | 80.6 | 74.5 | 83.2 | 92.0 | 81.4 | 89.1 | 80.6 | 86.2 | 82.5 | 90.8 |  |
| **CTL16-2018** | 81.8 | 89.8 | 80.4 | 69.2 | 82.8 | 91.1 | 81.1 | 87.9 | 80.6 | 86.4 | 82.5 | 90.6 |  |
| **CTL43-2018** | 81.0 | 88.8 | 80.6 | 73.9 | 80.5 | 85.2 | 80.9 | 87.9 | 80.2 | 85.2 | 82.4 | 90.6 |  |


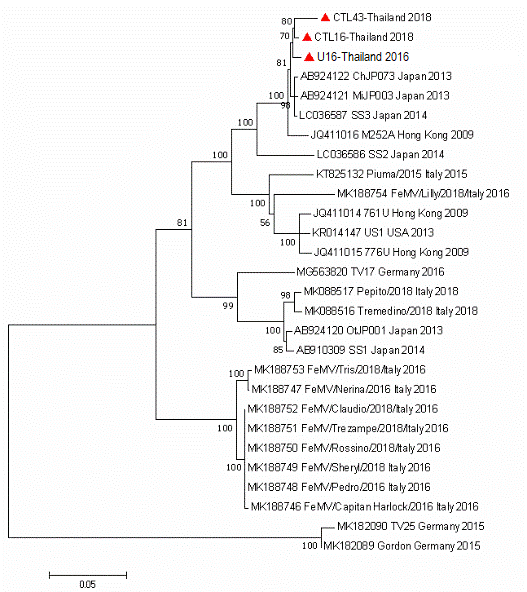

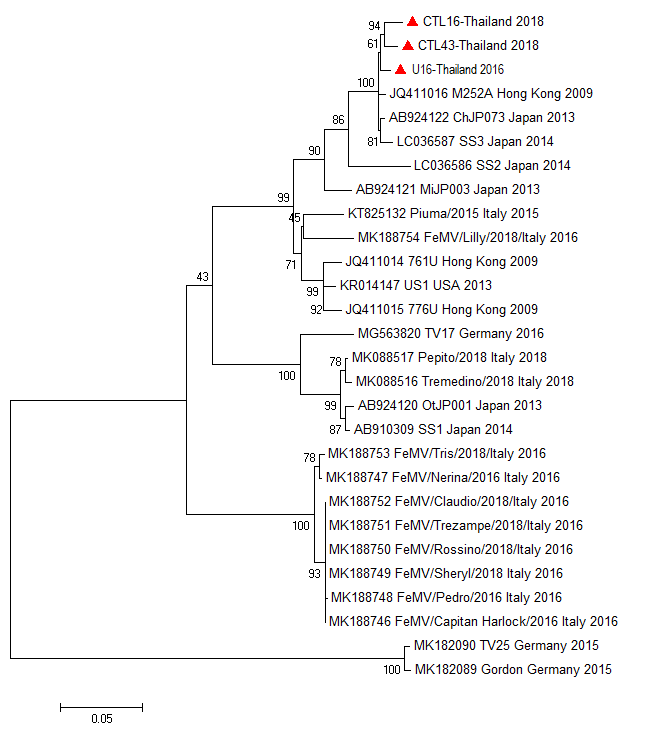


**H**

**P**

**F**

**N**


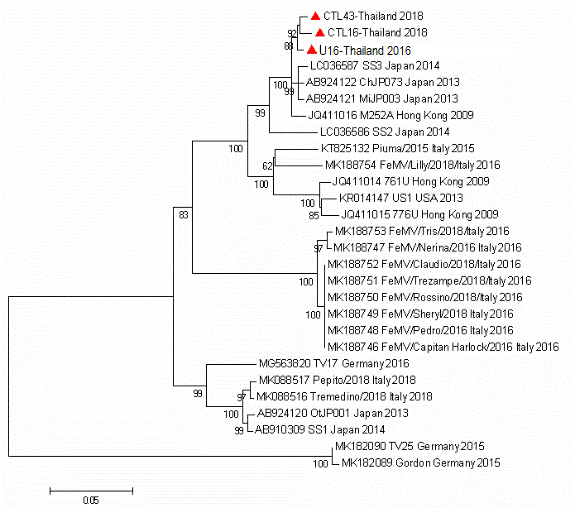

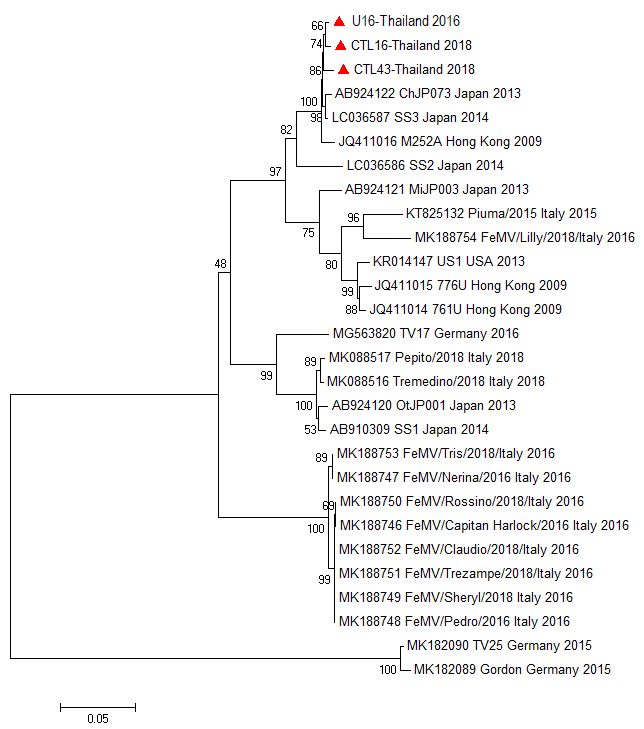

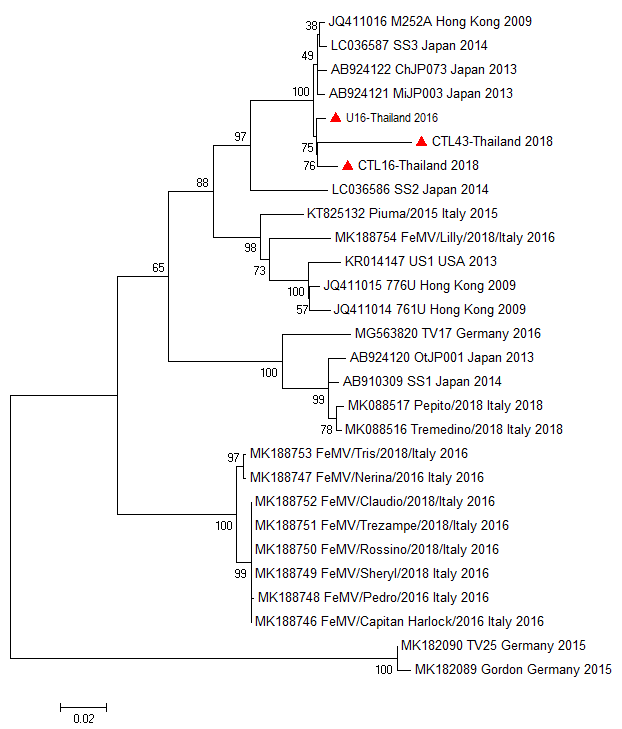

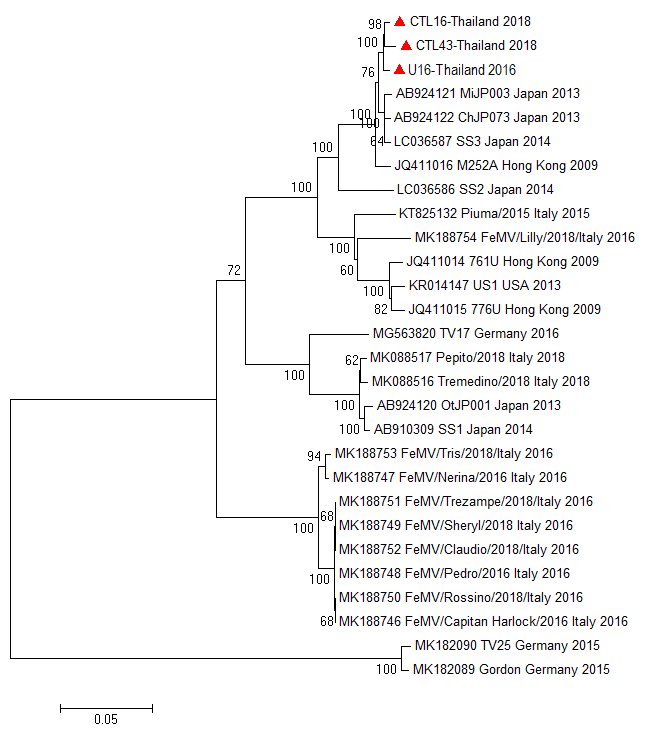


**L**

**M**

**Supplementary Figure S1**

Phylogenetic analysis of the codon region sequence of the six genes in FeMV strains.


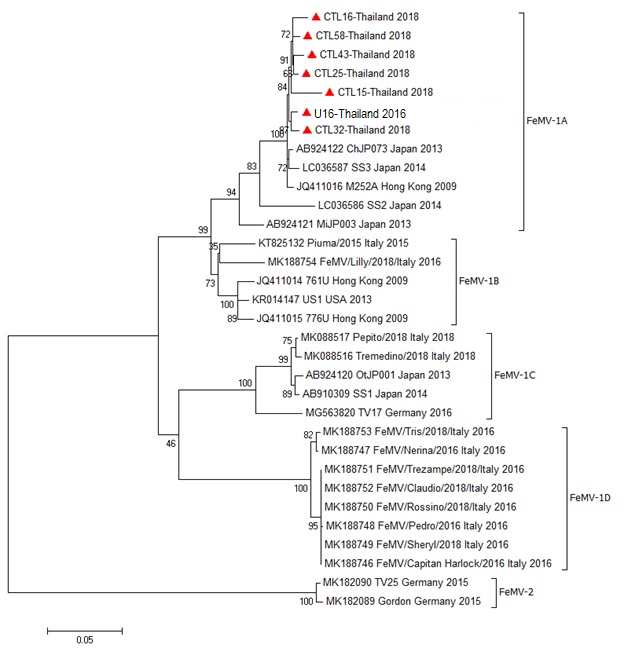


**Supplementary Figure S2**

Phylogenetic tree of the codon region of the F gene among FeMVs. Scale bar is the substitution rate per site. The ML method with a GTR model and 1000 bootstrap replicates (shown as a %) were performed in the Mega 7 software.

**
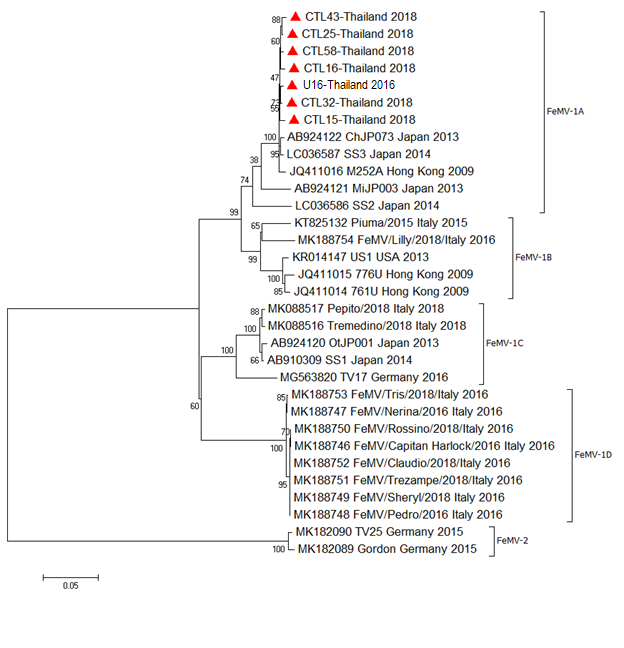
**

**Supplementary Figure S3**

Phylogenetic tree of the codon region of the H gene among FeMVs. Scale bar is the substitution rate per site. The ML method with a GTR model and 1000 bootstrap replicates (shown as a %) were performed in the Mega 7 software.
